# Combined use of murine double minute-2 promoter methylation and serum AFP improves diagnostic efficiency in hepatitis B virus-related hepatocellular carcinoma

**DOI:** 10.7150/ijms.47003

**Published:** 2020-10-23

**Authors:** Jing-Wen Wang, Yu Qian, Chen-Si Wu, Ning-Hui Zhao, Yu Fang, Xiao-Dong Yuan, Shuai Gao, Yu-Chen Fan, Kai Wang

**Affiliations:** 1 Department of Hepatology, Qilu Hospital of Shandong University, Jinan 250012, China.; 2 Institute of Hepatology, Shandong University, Jinan 250012, China.

**Keywords:** HBV-related HCC, MDM2, DNA methylation

## Abstract

**Objective:** Hepatocellular carcinoma (HCC) accounts for approximately 85% of all cases of liver cancer. In China, chronic hepatitis B virus-related HCC (HBV-related HCC) is the most common type of HCC. However, the majority of HBV-related HCC patients are asymptomatic, and the best opportunities for treating these patients are missed. The precise diagnosis of HBV-related HCC is crucial. The main purpose of this study was to evaluate the diagnostic value of murine double minute-2 (MDM2) promoter methylation in HBV-related HCC patients.

**Methods:** The methylation status of the MDM2 promoter was detected by methylation-specific PCR. The MDM2 expression levels were validated by quantitative real-time PCR. Enzyme-linked immunosorbent assay was used to determine the levels of interleukin-6 (IL-6) and tumor-necrosis factor-α (TNF-α) in plasma.

**Results:** The methylation frequency of the MDM2 promoter was decreased in HBV-related HCC patients. The MDM2 mRNA levels of patients with HBV-related HCC were higher than those of patients with liver cirrhosis and chronic hepatitis B. The plasma levels of IL-6 and TNF-α were significantly higher in HBV-related HCC patients than that in liver cirrhosis and chronic hepatitis B patients. The TNF-α levels were higher in the unmethylated MDM2 promoter group than in the methylated MDM2 promoter group in HBV-related HCC patients. Moreover, the combination of MDM2 promoter methylation and alpha-fetoprotein (AFP) improved the diagnosis of HBV-related HCC.

**Conclusions:** Our study indicates, for the first time, that MDM2 promoter hypomethylation is present in HBV-related HCC patients. The combination of MDM2 promoter methylation and AFP can greatly improve diagnostic efficiency in HBV-related HCC, which might provide a new method for HBV-related HCC diagnosis.

## Introduction

Hepatocellular carcinoma (HCC) is the major type of primary liver cancer, accounting for 85% of liver cancer cases worldwide 1. HCC is the third leading cause of death from malignant tumors in the world, and the development of HCC is closely related to alteration in the structure or expression of certain oncogenes 2-4. In China, chronic hepatitis B virus (HBV) infection is one of major causes of HCC 5. To date, alpha-fetoprotein (AFP) has been used as HCC marker for more than 50 years, but its sensitivity and specificity are limited. It is particularly important to improve the diagnostic efficiency of HBV-related HCC. Therefore, there is an urgent need to search for a potential biomarker to diagnose HBV-related HCC.

Murine double minute-2 (MDM2) is an E3 ubiquitin ligase, and the gene encodes a negative regulator of the p53 tumor suppressor 6. MDM2 binds to the transcriptional activation domain of p53 to regulate the stabilization and activation of p53 7,8. Under normal physiological conditions, MDM2 and p53 form a self-regulating negative feedback loop to ensure a balance between them 9. However, the negative feedback loop is often observed to be broken in various tumors. MDM2 overexpression, in particular, has been implicated in several tumors, such as sarcoma, colon cancer and gastric cancer 10-12. Previous studies have shown that MDM2 is an optimal target for therapy in tumors carrying wild-type tumor protein 53 13-15. Furthermore, MDM2 can regulate the target protein interleukin-6 (IL-6) and tumor-necrosis factor-α (TNF-α), which play an important role in regulating the mitogen-activated protein kinase (MAPK) and nuclear factor-kappa B (NF-κB) pathways 16.

Epigenetic modification, including aberrant DNA methylation, is one of the important events in carcinogenesis 17. DNA methylation is a heritable change in gene expression without DNA sequence change 18. DNA methylation is of great importance for gene expression and can have dire consequences for cells 19. The overexpressed oncoprotein MDM2 not only binds to p53 and negatively regulates p53 but also contributes to HCC development, regardless of p53 status. A previous study has suggested that MDM2 hypomethylation and overexpression take place in pterygium 20. DNA methylation has been widely investigated in a variety of tumors, such as gastric cancer and lung cancer 21,22. Multiple studies have demonstrated that DNA methylation of some genes can be used as biomarkers for diagnosis, prognosis or therapeutic strategies 23,24.

However, studies associating MDM2 methylation in diseases are scarce. To the best of our knowledge, the methylation status of MDM2 in HBV-related HCC has not yet been explored. In this study, we detected the methylation status of MDM2, and we quantitatively analyzed the mRNA levels of MDM2 in peripheral blood mononuclear cells (PBMCs). Moreover, we determined the IL-6 and TNF-α levels in plasma. We aimed to determine the value of MDM2 methylation in the diagnosis of HBV-related HCC.

## Materials and methods

### Subjects

One hundred patients with HBV-related HCC, 31 patients with liver cirrhosis (LC), and 37 patients with chronic hepatitis B (CHB) were recruited from June 2016 to February 2018 at the Department of Hepatology, Qilu Hospital of Shandong University. CHB patients were selected according to the practice guidelines for managing CHB established in the 2018 update of the American Association for the Study of Liver Diseases 25. The inclusion criteria for LC patients followed the evidence-based clinical practice guidelines for liver cirrhosis 2015 26. All HBV-related HCC patients were selected according to the 2010 update of the American Association for the Study of Liver Diseases Practice Guidelines for Management of hepatocellular carcinoma 27.

**Figure 1:**
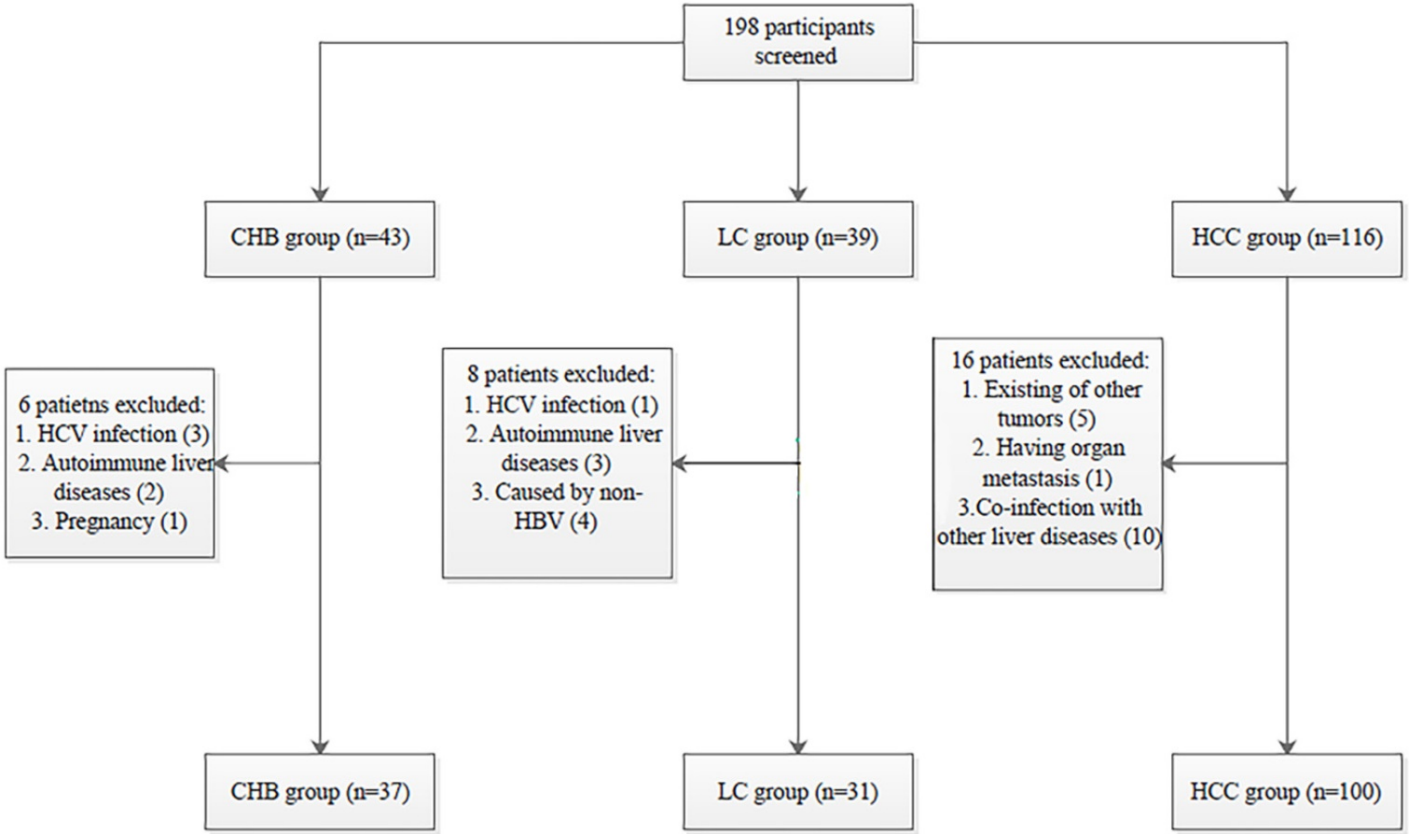
** Patient selection process.** HBV: hepatitis B virus; HCV: hepatitis C virus; CHB: chronic hepatitis B; LC: liver cirrhosis; HBV-related HCC: hepatitis B virus-related hepatocellular carcinoma.

All procedures performed in the studies involving human participants were in accordance with the Declaration of Helsinki and the ethical standards of the Medical Ethics Committee of Shandong University Qilu Hospital (NO. 2019058). Informed consent was obtained from all individual participants included in the study. The selection process of participants is shown in **Fig. 1.**

### Genomic DNA extraction and sodium bisulfite modification

Genomic DNA was extracted from PBMCs using the QIAamp DNA Mini Kit (Qiagen, Hilden, Germany). Genomic DNA modification was performed using EZ DNA Methylation-Gold kit (Zymo Research, Irvine, CA, USA), and the procedure was conducted in accordance with the instructions. Bisulfite-converted DNA (20 μL) was used for methylation-specific polymerase chain reaction (MSP) or stored in a refrigerator at -80 ºC for standby application.

### Methylation-specific PCR (MSP)

**Figure 2:**
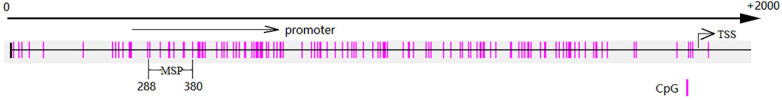
**MSP amplification regions and TSS.** MSP amplified regions (288-380) are indicated and TSS (1477) is indicated. MSP: methylation-specific PCR; TSS: transcription start site.

The modified DNA was amplified by MSP. The MDM2 promoter structure and MSP primer are shown in **Fig. 2.** The MDM2 primers for MSP were shown as follows: methylated forward primer 5'- TAACGGTTAAAGGAGTGTTATAGCG -3' and reverse primer 5'- GAAATAAAAATATTAACCGCGAAC -3'; unmethylated forward primer 5'-AATGGTTAAAGGAGTGTTATAGT-3' and reverse primer 5'-CAAAATAAAAATATTAACCACAAAC-3'. Each MSP contained 1 μL modified DNA, 0.5 μL forward and reverse primers (10 μM), 12.5 μL Premix Taq (Zymo Research, CA, USA), and 10.5 μL nuclease-free water. The PCR program included initial denaturation at 95 ºC for 5 min, followed by 35 cycles of 95 ºC for the 30 sec, 58.5 ºC for 30 sec, and 72 ºC for 30 sec; and a final extension at 72 ºC for 10 min. Water without DNA was used as the negative control. The separation of the 10 μL PCR products was conducted by electrophoresis on a 2% agarose gel, which was stained with Gel Red (Biotium, California, USA), and visualized under UV illumination. The MDM2 methylation status was determined positive if the MDM2 methylated primer or MDM2 methylated primer and MDM2 unmethylated primer both produced a band of expected size; otherwise it was negative.

### RNA extraction and quantitative real-time polymerase chain reaction (RT-qPCR)

Total RNA was extracted from 5 ml peripheral venous blood by TRIzol (Invitrogen, Carlsbad, CA, USA). The RNA was transcribed into cDNA using first-strand cDNA synthesis kit (Fermentas, Vilnius, Lithuania). The levels of MDM2 were measured by RT-qPCR, using the SYBR Green PCR mix (Takara, Japan), the Agilent Mx3005P (Agilent Technologies, USA). β-actin was used as the internal reference gene. The primer sequences were described as follows: MDM2: forward primer 5'-GGGAGTGATCAAAAGGAC-3' and reverse primer 5'-CCAAATGTGAAGATGAAGGTTTC-3' 28; β-actin: forward primer 5'-ATGGGTCAGAAGGATTCCTATGTG-3' and reverse primer 5'- CTTCATGAGGTAGTCAGTCAGGTC-3'. The total volume of 20 μL PCR solution contained 10 μL SYBR Green premix, 8.2 μL nuclease-free water, 1 μL cDNA and 0.4 μM of each primer. Each PCR program consisted of denaturation at 95 ºC for the 30 sec, followed by 40 cycles of 95 ºC for 5 sec, 60 ºC for 30 sec and 72 ºC for 30 sec. The mRNA level was determined using the comparative (2^-ΔΔCt^) method.

### Enzyme-linked immunosorbent assay (ELISA) for detection of IL-6 and TNF-α in plasma

Plasma was obtained from peripheral venous blood by centrifugation (3000 r/min, 10 min). The plasma IL-6 and TNF-α levels were measured using the Human IL-6 Immunoassay Quantikine ELISA kit (R&D San Diego, CA, USA) and Human TNF-α Immunoassay Quantikine ELISA kit (R&D San Diego, CA, USA) according to the manufacturer's instructions.

### Statistical analysis

Statistical analysis was performed using SPSS version 19.0 software (SPSS, Chicago, IL, USA) and GraphPad Prism 6.0 (San Diego, CA, USA). The quantitative variables were expressed as median (centile 25; centile 75), and the differences between groups were compared using Mann-Whitney U-test or Kruskal-Wallis test. The categorical variables were expressed as number (%), and differences between groups were compared with Chi-squared tests. Associations of the MDM2 expression levels with the clinicopathological characteristics of patients were analyzed by the Spearman correlation test. In order to assess the diagnostic value of MDM2 promoter methylation, we combined MDM2 promoter methylation and the AFP to measure the area under the receiver operating characteristic (ROC) curve. The results were considered statistically significant when *P*<0.05.

## Results

### Clinical characteristics

In this study, we included a total of 168 subjects. The clinicopathological variables are shown in **Table 1.** The clinical differences between the patients in the MDM2 methylated group and those in the MDM2 unmethylated group are shown in **Table 2.** The significant gender and HBe Ag differences between the patients with LC the MDM2 methylated group and MDM2 unmethylated group are shown. Among the CHB and LC patients, the AFP levels in the MDM2 unmethylated group were higher than those in MDM2 methylated group. However, there was no statistically significant difference in the patients with HCC.

### MDM2 promoter methylation is decreased in HBV-related HCC patients

**Figure 3:**
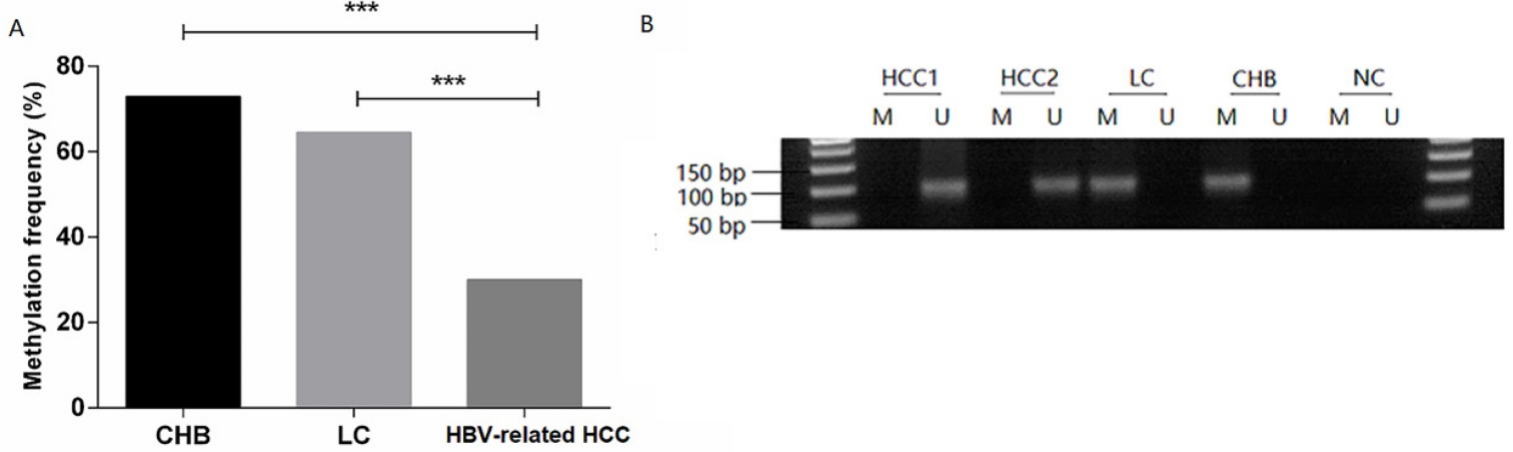
** MDM2 promoter methylation status in patients.** (A) MDM2 promoter methylation frequency of HBV-related HCC, LC, and CHB patients. (B) Methylation-specific PCR results of the MDM2 promoter. CHB: chronic hepatitis B; LC: liver cirrhosis; HBV-related HCC: hepatitis B virus-related hepatocellular carcinoma; M: methylated sequence; U: unmethylated sequence; NC: negative control. ***P<0.001.

The methylation frequency of MDM2 detected in PBMCs was 72.97% (27/37) in the CHB patients, 64.52% (20/31) in the LC patients, 30.00% (30/100) in the HBV-related HCC patients. The MDM2 methylation frequency in the HBV-related HCC patients was significantly lower than that in CHB patients [χ^2^=20.528, odds ratio (OR) = 6.300, 95% confidence interval (CI): 2.714-14.626, *P*=0.000] and LC (χ^2^=11.946, OR=4.242, 95%CI: 1.811-9.936, *P*=0.001) (**Fig. 3A**). However, no difference in the MDM2 methylation frequency was detected between the CHB and LC patients (χ^2^=0.563, *P*=0.452). The agarose gel electrophoresis results showing the MDM2 promoter methylation status are presented in **Fig. 3B.**

### MDM2 promoter methylation is associated with tumor node metastasis (TNM) stage and Barcelona Clinic Liver Cancer (BCLC) stage

Next, we analyzed the correlation between MDM2 promoter methylation and clinicopathological characteristics in the HBV-related HCC patients (**Table 3**). The results revealed that MDM2 promoter methylation was significantly correlated with distant metastasis (*P*=0.040), TNM stage (*P*=0.035) and BCLC stage (*P*=0.044). There was no correlation between MDM2 promoter methylation and other clinicopathological characteristics, such as gender, age, AFP, vascular invasion, lymph node metastasis, number of tumors, tumor size.

### Association between mRNA levels of *MDM2* in PBMCs and clinicopathological characteristics

**Figure 4:**
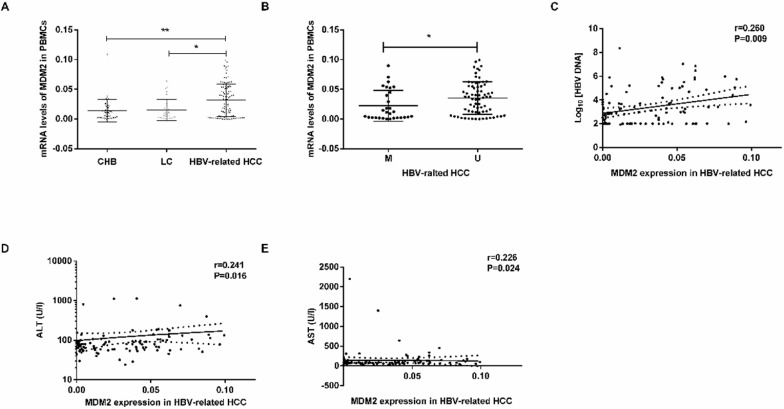
** MDM2 mRNA level and its relationship with clinicopathological characteristics.** (A) The mRNA levels of MDM2 among different samples from CHB, LC and HBV-related HCC. (B) MDM2 mRNA levels in HBV-related HCC patients in MDM2 M group and U group. (C-E) The linear correlation between MDM2 mRNA levels and clinicopathological characteristics in HBV-related HCC patients. CHB: chronic hepatitis B; LC: liver cirrhosis; HBV-related HCC: hepatitis B virus-related hepatocellular carcinoma; M: methylated group; U: unmethylated group; ALT: alanine aminotransferase; AST: aminotransferase aspartate. *P<0.05, **P<0.01.

MDM2 mRNA levels were obviously higher in the HBV-related HCC patients than in the CHB patients (*P*=0.0034) LC patients (*P*=0.0102) (**Fig. 4A**). There were no significant differences in MDM2 mRNA levels between the CHB and LC patients (*P*>0.05). In the HBV-related HCC patients, the MDM2 mRNA levels in the unmethylated group were significantly higher than those in the methylated group (*P*=0.0236, **Fig. 4B**).

We next studied the correlation between MDM2 mRNA levels and clinicopathological characteristics in the HBV-related HCC patients. We found a positive correlation between MDM2 mRNA levels and HBV DNA (P=0.009), alanine aminotransferase (ALT) (P=0.016), aminotransferase aspartate (AST) (P=0.024) (**Fig. 4C-E**). However, there was no correlation between MDM2 mRNA levels and total bilirubin (TBIL), albumin (ALB) or prothrombin time (PT).

### Levels of IL-6 and TNF-α are increased in plasma of HBV-related HCC patients

**Figure 5:**
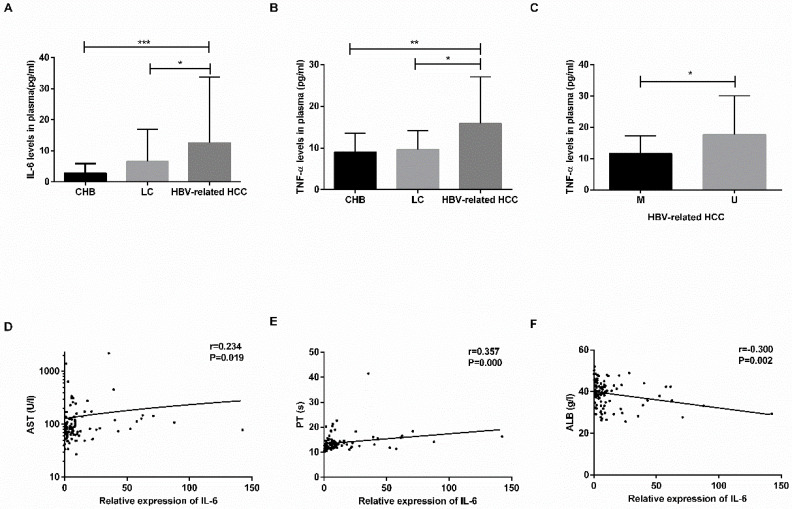
** The concentration of IL-6 and TNF-α in plasma and relationship with clinicopathological characteristics.** (A-B) The plasma levels of IL-6 and TNF-α in CHB, LC, HBV-related HCC patients. (C) The difference in TNF-α levels in HBV-related HCC patients in the M group and U group. (D-F) The linear correlation between IL-6 levels and clinicopathological characteristics of HBV-related HCC patients. CHB: chronic hepatitis B; LC: liver cirrhosis; HBV-related HCC: hepatitis B virus-related hepatocellular carcinoma; IL-6: interleukin-6; TNF-α: tumor-necrosis factor-α; M: methylated group; U: unmethylated group; AST: aminotransferase aspartate; PT: prothrombin time; ALB: albumin. *P<0.05, ** *P*<0.01, *** *P*<0.001.

The plasma levels of IL-6 and TNF-α are shown in **Table 4.** The levels of IL-6 and TNF-α were increased in the HBV-related HCC patients compared with the CHB patients (IL-6: *P*=0.0001; TNF-α: *P*=0.0031) and LC patients (IL-6: *P*=0.0171; TNF-α: *P*=0.0176) (**Fig. 5A-B**). No significant difference was found between the CHB and LC patients (*P*>0.05).

In the HBV-related HCC patients, the TNF-α levels in the MDM2 unmethylated group were significantly increased in the MDM2 methylated group (*P*=0.0246) (**Fig. 5C**), the levels of IL-6 in the MDM2 unmethylated group were slightly higher than those in the MDM2 unmethylated group (*P*>0.05). The IL-6 levels were positively correlated with AST (*P*=0.019) and PT (*P*=0.000) in the HBV-related HCC patients (**Fig. 5D-F**) but were negatively correlated with ALB (*P*=0.002) (**Fig. 5E**). We did not find any possible relationship between TNF-α and clinicopathological features.

### Combination of *MDM2* promoter methylation and AFP can improve diagnostic efficiency in HBV-related HCC

**Figure 6:**
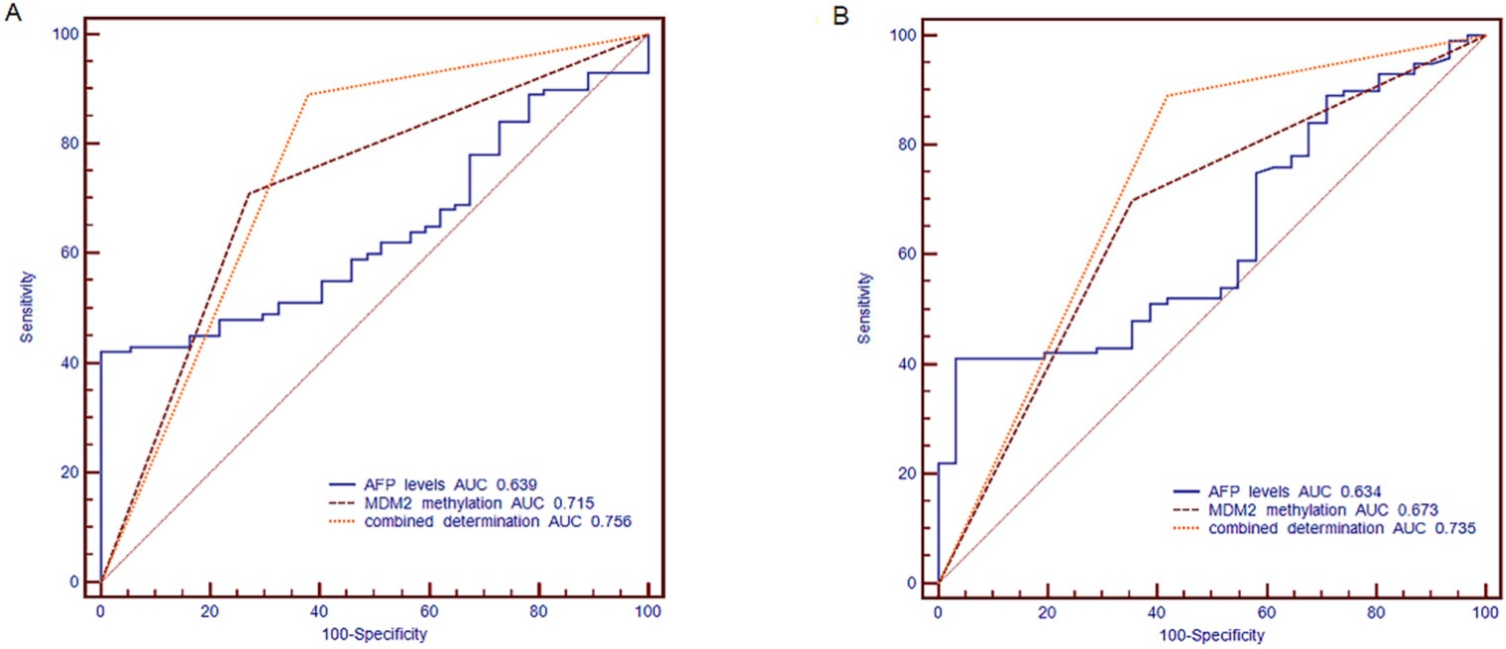
** The receiver operating characteristic (ROC) curve of MDM2 promoter methylation and serum AFP in discriminating HBV-related HCC from CHB and LC patients.** (A) Discriminating HBV-related HCC from CHB patients. (B) Discriminating HBV-related HCC from LC patients. AFP: alpha-fetoprotein; AUC: area under curve.

The ROC curve was established, including AFP, methylation status of MDM2 and combined determination, to identify the HBV-related HCC, CHB and LC. We compared the diagnostic value of AFP (cut off point: 20 ng/ml), MDM2 methylation, and combined determination to distinguish HBV-related HCC from CHB. For the combination determination, the sensitivity was 89.00%, the specificity was 62.16%, the positive predictive value (PPV) was 87.25%, and the negative predictive value (NPV) was 62.07%, which was better than AFP (sensitivity: 52%, specificity: 62.16%, PPV: 78.79%, NPV: 32.39%) and MDM2 promoter methylation (sensitivity: 70.00%, specificity: 72.97%, PPV: 87.5%, NPV: 43.37%). Moreover, the area under curve (AUC) of the combined determination was 0.756 [standard error (SE)=0.0434, 95% CI=0.675-0.825)], which was significantly higher than AFP levels (AUC=0.639, SE=0.0471, 95% CI=0.553-0.720; *P*=0.0056) and slightly higher than MDM2 promoter methylation (AUC=0.715, SE=0.0436, 95% CI=0.632-0.789; *P*>0.05) (**Fig. 6A**).

Meanwhile, AFP (cut off point: 20 ng/ml) combined with MDM2 methylation in distinguishing the HBV-related HCC patients from LC patients showed a sensitivity of 89.00%, specificity of 58.06%, PPV of 87.25%, and NPV of 62.07%, which were higher than the results of AFP (sensitivity: 52.00%, specificity: 58.06%, PPV: 80.00%, NPV: 27.27%) and MDM2 methylation (sensitivity: 70%, specificity: 64.52%, PPV: 86.42%, NPV: 40.00%). The combined determination AUC was 0.735 (SE=0.0477, 95% CI=0.651-0.809], which was higher than that of AFP alone (AUC=0.634, SE=0.0533, 95% CI=0.545-0.716; *P*=0.0128) and MDM2 methylation (AUC=0.673, SE=0.0494, 95% CI=0.585-0.752; *P*=0.0356) (**Fig. 6B**).

## Discussion

Early-stage HCC is usually asymptomatic, and only 20% of cases of HCC are surgically removed 29. Epigenetic alterations, particular DNA methylation, could be used for assessment in diagnosis 30. In the present study, we first found that MDM2 promoter methylation existed in HBV-related HCC patients, LC patients and CHB patients. In addition, the MDM2 promoter methylation frequency in HBV-related HCC patients was lower than that in LC patients and CHB patients. MDM2 promoter methylation combined with AFP could be used as an effective diagnostic method for HBV-related HCC.

Oncogene activation, tumor suppressor function attenuation and inflammatory responses are involved in the pathogenesis of HCC. DNA methylation might be linked to the pathogenesis of HCC. MDM2 is an oncogene that can be expressed in PBMCs 31,32. It is known that the products of MDM2 gene play an important role in HCC and promote tumor growth 33. Previous study indicated that MDM2 gene promoter hypomethylation and overexpression occur in patients with pterygium 20. We found that the methylation frequency of the MDM2 promoter in patients with HBV-related HCC was significantly decreased compared to that in LC and CHB patients. The decreased MDM2 promoter methylation showed a correlation with distant metastasis, TNM stage and BCLC stage in patients with HCC. These finds suggest that MDM2 gene promoter hypomethylation takes place in HBV-related HCC patients and is involved in HBV-related HCC progression. The exact mechanism of DNA methylation is not clear, several studies demonstrate that there is a correlation between oxidative stress and DNA methylation 34,35. Oxidative stress could cause oncogene demethylation, which leads to oncogene overexpression and might play a role in the occurrence and development of HCC 36. Our previous research has pointed out that IGFBP7 gene promoter methylation is related to oxidative stress in HBV-related HCC patients, while oxidative stress is involved in the development and progression of HCC 37. We will make further study on the relationship between MDM2 gene promoter methylation and oxidative stress in the next research work.

MDM2 overexpression leads to loss of p53 function, resulting in the occurrence of tumors 38. In humans, MDM2 overexpression is common in many different tumor types, such as soft tissue tumor, osteosarcoma and esophageal cancer 39. Gene promoter methylation can lead to repressions of transcription. In this study, we showed that HBV-related HCC patients have increased MDM2 mRNA levels. This result is compatible with previous study by Zhang et al 40. We also found that MDM2 mRNA levels were negatively correlated with MDM2 gene promoter methylation status in HBV-related HCC. This finding confirms a previous study that gene promoter methylation usually leads to transcriptional silencing 41. However, it has already known that there is a mechanism of p53 degradation that is not mediated by MDM2 42. E3 ubiquitin ligases (E3s) can be divided into the really interesting new genes (RING)-finger protein family and the homologous to E6AP carboxyl terminus (HECT) family. MDM2 and Pirh2 belong to the single-subunit RING finger E3s, COP1 belongs to the subunit of multiunit RING finger E3s, as well as Arf-BP1 belongs to the HECT family. Previous studies reported elevated levels of MDM2, Pirh2, COP1 and Arf-BP1 in HCC and other tumors compared with those in normal tissues 43-46. COP1 and Pirh2 are negative regulators of p53 by ubiquitination independent of MDM2 and could be the predictors for HCC survival 47,48. Arf-BP1 is also an E3 ligase of the p53-independent and p53-dependent *in vivo* 49. Although these E3 ligases are involved in MDM2-independent p53 degradation, the roles of these ligases in the regulation of p53 and the usefulness in the diagnosis of HCC remain to be explored.

MDM2 promoted the production of TNF-α and IL-6 through the MAPK and NF-κB pathways 16. IL-6 and TNF-α play an important role in the occurrence and development of tumors, including HCC 50. The occurrence and poor prognosis of HCC are associated with the higher serum levels of IL-6 51,52. Elevated TNF-α levels are found in several cancer tissues, such as ovarian and renal cancers 50,53. Blocking MDM2 has anti-inflammatory and anti-cancer effects 54-56. In this present study, we observed that plasma IL-6 and TNF-α levels were significantly higher in the HBV-related HCC patients. The TNF-α levels were lower in the methylated MDM2 promoter group than those without. These results indicate a link between MDM2 gene promoter methylation and inflammatory factors elevation in HBV-related HCC.

Serum AFP has been used in the diagnosis of HCC, however, serum AFP is often elevated in inflammation and other non-cancerous conditions; in addition, 40% of HCC patients do not manifest an elevation in serum AFP 57,58. The promoter methylation of certain genes plays an important role in human cancers, these genes can be used as diagnostic biomarkers. In this study, we constructed the ROC curve to investigate the diagnostic value of combination MDM2 promoter methylation and AFP. The results indicated that the sensitivity and specificity of combining AFP and MDM2 methylation were higher compared to AFP alone. We also found that the decreased MDM2 promoter methylation correlates with the early stage of HBV-related HCC. These results suggested the method of diagnosing cancer that combined of AFP and MDM2 promoter methylation could be used to diagnose early HBV-related HCC. The present study first revealed the crucial value of the combination of MDM2 promoter methylation and serum AFP in the early diagnosis of HBV-related HCC. Sorafenib is a tyrosine kinase inhibitor and has a significant therapeutic effect on advanced HCC. It has been shown that circulating cell-free DNA may be potential biomarkers for determining the effect of HCC treatment 59. The predictive value of MDM2 methylation for the treatment outcome of sorafenib will be performed in the future study.

There are some limitations to this research. First, our study was a retrospective single-center study. Further external validation with a large-sample, multiple-center is needed to verify the role of MDM2 promoter methylation as a biomarker. We should monitor the diagnostic value of MDM2 methylation. Second, there was a lack of liver tissues, therefore, we only performed this study in PBMCs. Third, we did not find alterations in MDM2 methylation in the CHB or LC patients from non-cancerous to cancerous diseases. However, the current report revealed the non-invasive early detection of HCC four years before conventional diagnosis using methylation biomarkers 60. These results showed the great potential of methylation biomarker for predicting cancerous diseases in non-cancerous diseases. Therefore, whether unmethylated patients with CHB or LC are having risk should be verified in prospective cohort in the future. In addition, we only found that the MDM2 promoter methylation is a trend in HBV-related HCC, but the specificity of MDM2 promoter methylation for HBV-related HCC or non-HBV-related HCC should be noted and performed in the future study.

## Conclusions

In conclusion, this study showed MDM2 promoter hypomethylation in HBV-related HCC patients. Moreover, the combination of MDM2 promoter methylation and serum AFP might improve the diagnostic efficiency of HBV-related HCC.

## Figures and Tables

**Table 1:** Baseline characteristics of the patients

Variable	CHB (n=37)	LC (n=31)	HCC (n=100)	*P* value
Gender (M/F)	25/12	24/7	80/20	0.309^b^
Age (years)	41 (31-53)	48 (39-55)	54 (49-62)	**<0.001^a^**
Log_10_[HBV DNA]	6.33 (4.29-7.04)	4.90 (3.52-7.14)	2.94 (2.00-4.35)	**<0.001^ a^**
HBe Ag (+/-)	26/11	20/11	30/70	**<0.001^ b^**
ALT (U/I)	88 (58.00-101.50)	71.00 (48.00-97.00)	78.50 (59.00-103.25)	0.597^ a^
AST (U/I)	84.00 (53.00-116.50)	63.00 (50.00-90.00)	82.50 (65.25-120.75)	**0.048^ a^**
TBIL (μmol/L)	18.70 (12.10-45.35)	37.20 (18.30-83.80)	23.95 (16.55-52.08)	0.106^ a^
ALB (g/L)	42.40 (38.85-45.30)	38.60 (28.70-42.40)	39.90 (34.83-43.05)	**0.015^ a^**
PT (s)	12.10 (11.40-13.55)	13.90 (12.70-16.70)	13.10 (11.83-14.78)	**<0.001^ a^**
AFP (ng/ml)	12.75 (3.83-33.26)	16.82 (2.83-83.44)	28.41 (5.99-1214.65)	**0.010^ a^**
**Child-Pugh class**				0.619 ^a^
A	NA	16	61	
B	NA	11	27	
C	NA	4	12	
**Antiviral agent**				0.604 ^a^
Adefovir	10	6	27	
Entecavir	17	18	52	
Tenofovir	1	2	1	
Lamivudine	1	2	3	
No	8	3	17	
**Treatment duration**				0.582 ^a^
< 6 months	18	16	40	
6-12 months	3	1	12	
12-24 months	2	2	10	
> 24 months	6	9	21	
MDM2 methylation	27 (72.97%)	20 (64.52%)	30 (30.00%)	**<0.001^ b^**

CHB: chronic hepatitis B; LC: liver cirrhosis; HCC: hepatocellular carcinoma; HBe Ag: hepatitis B e antigen; ALT: alanine aminotransferase; AST: aminotransferase aspartate; TBIL: total bilirubin; ALB: albumin; PT: prothrombin time; NA: not available. a: Kruskal-Wallis H test. b: Chi-square test.

**Table 2:** The clinical differences between patients in the MDM2 methylated group and the MDM2 unmethylated group

Variable	CHB (n=37)		*P* value	LC (n=31)		*P* value	HBV-related HCC (n=100)		*P* value
	Methylated	Unmethylated		Methylated	Unmethylated		Methylated	Unmethylated	
Gender (M/F)	17/10	8/2	0.326^b^	13/7	11/0	**0.026^ b^**	24/6	56/14	1.000^b^
Age (years)	41 (31-54)	48.5 (31.75-52.5)	0.768^a^	48 (40.5-55.75)	44 (31-55)	0.364^ a^	52.5 (46.75-63)	54 (50-62)	0.377^a^
Log_10_[HBV DNA]	6.03 (4.16-7.17)	6.39 (4.13-7.06)	>0.05^a^	4.61 (3.02-7.05)	6.68 (4.18-7.42)	0.182^ a^	2.84 (2.00-4.69)	3.04 (2.00-4.24)	0.977^a^
HBe Ag (+/-)	21/6	5/5	0.101^ b^	10/10	10/1	**0.023^b^**	7/23	23/47	0.341^b^
ALT (U/I)	81.00 (57.00-101.00)	90.00 (49.25-103.50)	0.756^ a^	61.50 (41.75-96.75)	84.00 (59.00-100.00)	0.256^ a^	75.00 (58.25-98.50)	79.00 (59.00-105.25)	0.566^a^
AST (U/I)	84.00 (54.00-117.00)	76.00 (48.75-111.25)	0.794^ a^	62.00 (47.50-88.75)	81.00 (50.00-113.00)	0.256^ a^	91.00 (69.25-138.50)	82.00 (64.75-114.75)	0.511^a^
TBIL (μmol/L)	16.70 (10.50-39.80)	24.70 (15.85-123.60)	0.253^ a^	25.95 (16.50-75.35)	47.70 (20.60-109.5)	0.208^ a^	20.70 (16.88-49.25)	24.95 (16.48-59.98)	0.743^a^
ALB (g/L)	42.80 (40.40-45.10)	39.60 (37.10-45.85)	0.473^a^	40.35 (29.98-42.30)	35.90 (28.70-43.10)	0.847^ a^	38.80 (34.78-41.90)	40.40 (34.50-43.95)	0.227^a^
PT (s)	12.10 (11.20-13.60)	12.00 (11.55-13.70)	0.743^ a^	13.70 (12.58-16.93)	14.40 (12.70-16.60)	0.677^ a^	13.45 (12.38-14.60)	12.70 (11.80-14.43)	0.124^a^
AFP (ng/ml)	7.44 (2.77-15.48)	43.54 (30.86-69.95)	**<0.001**^a^	4.56 (1.95-16.61)	91.05 (46.27-297.30)	**<0.001**^a^	93.63 (6.34-1719.69)	16.30 (5.84-1213.87)	0.528^a^

CHB: chronic hepatitis B; LC: liver cirrhosis; HBV-related HCC: hepatitis B virus-related hepatocellular carcinoma; HBe Ag: hepatitis B e antigen; ALT: alanine aminotransferase; AST: aminotransferase aspartate; TBIL: total bilirubin; ALB: albumin; PT: prothrombin time; AFP: alpha-fetoprotein. a: Kruskal-Wallis H test. b: Chi-square test.

**Table 3:** Association between clinicopathological features and the methylation of MDM2 promoter in patients with HBV-related HCC

Variable	Total number	Methylated	Unmethylated	*P* value
**Gender**				1.000
Male	80	24 (30.00%)	56(70.00%)	
Female	20	6 (30.00%)	14 (70.00%)	
**Age**				0.055
≥50	73	18 (24.66%)	55 (75.34%)	
<50	27	12 (44.44%)	15 (55.56%)	
**AFP (ng/ml)**				0.239
≥20	51	18 (35.29%)	33 (64.71%)	
<20	49	12 (24.49%)	37 (75.51%)	
**Vascular invasion**				0.752
Yes	22	6 (27.27%)	16 (72.73%)	
No	78	24 (30.77%)	54 (69.23%)	
**Lymph node metastasis**				0.399
Yes	22	5 (22.73%)	17 (77.27%)	
No	78	25 (32.05%)	53 (67.95%)	
**Distant metastasis**				**0.040**
Yes	9	0 (0.00%)	9 (100.00%)	
No	91	30 (32.97%)	61 (67.03%)	
**Number of tumors**				0.784
Multiple	51	13 (25.49%)	37 (74.51%)	
Solitary	49	17 (34.69%)	33 (65.31%)	
**Tumor size (cm)**				0.961
>5	27	8 (29.63%)	19 (70.37%)	
≤5	73	22 (30.14%)	51 (69.86%)	
**TNM stage**				**0.035**
Ι+ΙΙ	61	23 (37.70%)	38 (62.30%)	
ΙΙΙ+ΙV	39	7 (17.95%)	32 (82.05%)	
**BCLC stage**				**0.044**
A-C	86	29 (33.72%)	57 (66.28%)	
D	14	1 (7.14%)	13 (92.86%)	

MDM2: murine double minute-2; HCC: hepatocellular carcinoma; AFP: α-fetoprotein; TNM: tumor node metastasis; BCLC: Barcelona Clinic Liver Cancer. Statistical analysis was performed using Chi-square test.

**Table 4:** Plasma levels of IL-6 and TNF-α in patients

Cytokines (pg/ml)	CHB	LC	HBV-related HCC
IL-6	2.17 (0.89-3.62)	2.25 (0.64-8.20)	5.68 (2.13-10.43)
TNF-α	7.76 (5.98-12.24)	9.15 (5.37-13.26)	13.90 (9.36-19.96)

CHB: chronic hepatitis B; LC: liver cirrhosis; HBV-related HCC: hepatitis B virus-related hepatocellular carcinoma; IL-6: interleukin-6; TNF-α: tumor-necrosis factor-α.

## Abbreviations

MDM2: murine double minute-2
AFP: alpha-fetoprotein
CHB: chronic hepatitis B
LC: liver cirrhosis
HBV-related HCC: hepatitis B virus-related hepatocellular carcinoma
IL-6: interleukin-6
TNF-α: tumor-necrosis factor-α
